# Each to their own beat: periodicity in temporal inference

**DOI:** 10.1186/1471-2202-12-S1-P257

**Published:** 2011-07-18

**Authors:** Asma Motiwala, Charles Fox, Tony Prescott

**Affiliations:** 1 Department of Psychology, University of Sheffield, Sheffield, S10 2TN, UK

## 

Characterizing cortical computation has been a key challenge in neuroscience. It has been suggested that the cerebral cortex is involved in unsupervised learning [1] and the notion that cortical computation is probabilistic is being increasingly accepted [2]. It has also been shown that cortical regions are hierarchically connected [3] and this has important implications on the way information could be represented. Moreover, the classical notion that processing in the cortex is predominantly governed by its feedforward inputs has been largely replaced by the view that feedback signals and other contextual information play a crucial role in the way information is encoded and learnt [2,4,5]. There is increasing emphasis on the influence of ongoing activity [5], ie. the state of the network on the effects of feedforward inputs.

The hierarchical temporal memory (HTM) model of cortical computation [6] was used as the starting point for the current work. HTM is a hierarchical probabilistic model that uses spatial and temporal characteristics of its inputs to represent 'invariant causes' in the world. The key assumptions in the model are that learning in the cortex involves learning sequences of coincident patterns in its inputs and that the temporal patterns in inputs are critical for the brain’s ability to learn and infer with ambiguous sensory information.

For the current work we are investigating tactile perception and implementing the HTM model using sensory inputs from artificial whiskers. The HTM model emphasizes that temporal patterns in inputs are extremely important features for learning and inference in the brain. This is especially the case with tactile perception since very little information is available at any single instant; how sensory inputs evolve over time conveys indispensable information about stimuli in the world. In modeling unsupervised learning, ‘time’ is commonly treated as indexing sequential inputs [7]. However, we are suggesting that learning, inference and prediction with temporal patterns pose a theoretical need to model some mechanism to encode time not only in terms of a temporal succession of events and causes, but also as a metric of periodicity. This theoretical need is perfectly aligned with well established empirical evidence that the brain engages in a plethora of oscillations at several different scales and frequencies. Our implementation of HTM incorporates a mechanism which attempts to mimic hypothesized effects of local neural oscillations in temporal inference.

We are suggesting that cortical networks can dynamically maintain local oscillations to best match the relative frequencies with which inputs tend to occur. Assuming that all inputs to any cortical network are superimposed on its ongoing activity, these local oscillations can discretize the continuous stream of sensory inputs in signaling to subsequent levels in the hierarchy, as well as modulate the signal to suppress or enhance effects of specific components in the inputs based on their timing. We are demonstrating these effects in our implementation of HTM.
